# Syphilis in pregnant women and congenital syphilis: spatial pattern and relationship with social determinants of health in Mato Grosso

**DOI:** 10.1590/0037-8682-0316-2020

**Published:** 2020-10-21

**Authors:** Leila Regina de Oliveira, Emerson Soares dos Santos, Francisco José Dutra Souto

**Affiliations:** 1 Universidade Federal de Mato Grosso, Instituto de Ciências da Saúde, Sinop, MT, Brasil.; 2 Universidade Federal de Mato Grosso, Departamento de Geografia, Cuiabá, MT, Brasil.; 3 Universidade Federal de Mato Grosso, Faculdade de Medicina, Cuiabá, MT, Brasil.; 4 Faculdade de Ciências Biomédicas de Cacoal, Curso de Medicina, Cacoal, RO, Brasil.

**Keywords:** Syphilis, Medical record linkage, Spatial analysis, Space-time clustering, Social determinants of health

## Abstract

**INTRODUCTION:**

The increasing incidence of syphilis among pregnant women (PS) and congenital syphilis (CS) has negatively affected maternal-child health in Brazil. The spatial approach to diseases with social indicators improves knowledge of health situations. Herein, we aimed to evaluate the spatiotemporal distribution of incidences, identify the priority areas for infection control actions, and analyze the relationship of PS and CS clusters with social determinants of health in Mato Grosso.

**METHODS:**

This is an ecological study with data from different health information systems. After data procedure linkage, we analyzed the Bayesian incidences of triennial infections during specific periods. We performed SATSCAN screenings to identify spatiotemporal clusters. Further, we verified the differences between the clusters and indicators using Pearson’s chi-square test.

**RESULTS:**

The variations in PS incidence were 0.9-20.5/1,000 live births (LB), 0.6-46.3/1,000 LB, and 2.1-23.2/1,000 LB in the first, second, and last triennium, respectively; for CS, the variations were 0-7.1/1,000 LB, 0-7.5/1,000 LB, and 0.3-10.8/1,000 LB in the first, second, and last triennium, respectively. Three clusters each were identified for PS (RR=2.02; RR=0.30; RR=21.45, p<0.0001) and CS (RR=3.55; RR=0.10; RR=0.26, p<0.0001). The high-risk clusters overlapped in time-space; CS incidence was associated with municipalities with a higher proportion of LB mothers of race/non-white color and with poor sanitary conditions, lower proportion of pregnant teenagers, and under 8 years of schooling.

**CONCLUSIONS:**

The increase in the spatiotemporal evolution of PS and CS incidences and the extension of areas with persistent infections indicate the need for monitoring, especially of priority areas in the state.

## INTRODUCTION

Syphilis is a sexually transmitted disease caused by *Treponema pallidum* ^1^ ^,^ ^2^ that has been prevalent for several centuries, and its incidence in several countries has increased in recent years^3^. Syphilis is often asymptomatic and can be transmitted vertically to the conceptus, and can cause severe morbidity and mortality^1^ ^,^ ^2^.

In 2012, an estimated 900,000 cases of syphilis among pregnant women (PS) were reported globally, and resulted in more than 350,000 undesirable events, including 200,000 stillbirths and neonatal deaths^3^. An epidemic alert for congenital syphilis (CS) was issued in Brazil in 2016^4^, when estimates indicated approximately 18,000 cases in the previous year^5^.

The severity of the epidemiological situation of CS persists in Brazil, with more than 24,000 new cases reported in 2017^6^. In the same year, the incidences of PS and CS in the country were estimated at 17.2 and 8.6 per 1,000 live births (LB), respectively^6^. The incidence of CS exceeded the acceptable level (0.5 per 1,000 LB) at which mother-to-child transmission of syphilis can be certifiably eliminated^3^.

The spatial analysis of disease occurrence was performed to understand the health situation and to identify the population areas with greater and lesser vulnerabilities, which would facilitate the control and elimination of transmission, especially during epidemics^7^ ^,^ ^8^.

The disease distribution in a population does not occur randomly in space; the influence of various social determinants of health (SDH) is a critical determinant. SDH affects the health of individuals and groups in the population based on social, economic, psychological, cultural, behavioral, and ethnoracial factors that characterize different living environments, such as profession, housing, or leisure^9^. An analysis of the health situation based on the spatial distribution of diseases and socioeconomic indicators allows the planning of health actions and practices in services while targeting better care and distribution of financial resources. This could help achieve better health outcomes for the most vulnerable populations^10^ ^,^ ^11^.

In Mato Grosso, PS and CS incidences were estimated at 7.2/1,000 LB and 3.7/1,000 LB in 2016, which represented an increase of 157% and 208% respectively, compared to the incidences recorded in 2008^6^. Considering the significant impact of syphilis on maternal and child health, this study was performed to evaluate the spatiotemporal distribution of incidences and to identify priority areas for prevention, control, and elimination. The relationship between the clusters for CS, PS, and SDH in Mato Grosso were also analyzed.

## METHODS

This is an ecological study that aimed to illustrate the distribution of incidences, identify the areas of greatest risk, and analyze the relationship between socioeconomic indicators, including PS and CS clusters. Temporal analysis was performed for cases reported between January 1, 2008, and December 31, 2016. The units of analysis were 141 municipalities of Mato Grosso.

Mato Grosso is in the Midwest region of Brazil and is the third Brazilian state in territorial extension (906,807 km^2^)^12^. In 2018, the estimated population was 3,441,998, with a demographic density of 3.36 individuals per km^2^, a human development index (HDI) of 0.725 (11^th^ place in the Brazilian ranking), and Gini index of 0.469^12^. The number of LB was 53.531^13^, and the gross domestic product (GDP) per capita was 37,462.74 reals (4^th^ position in the Brazilian ranking) in 2016^14^.

To identify possible underreported cases in the Notifiable Diseases Information System (Sistema de Informação sobre Agravos de Notificação - SINAN), deterministic linkage was performed between PS and CS databases registered in SINAN, the Mortality Information System (Sistema de Informações sobre Mortalidade - SIM), and the Brazilian National Health System (SUS) Hospital Information System (SIH/SUS) using Stata software (version 12.0, StataCorp, College Station, TX, USA).

The local empirical Bayesian model was used to estimate the incidences of PS and CS in a less unstable manner, with random fluctuations smoothing the possible occurrence of cases. For each area, the rate was revalued by a weighted average between the measured value and the average rate of its neighborhood^15^. The incidences were estimated based on the full period data (2008-2016) and the trienniums of diagnosis (2008-2010, 2011-2013, and 2014-2016). The population under risk was considered the total number of LB in the same period and place of residence expressed per 1,000 LB.

The incidences of PS and CS were organized into five different layers for each infection, such that the first strata of each disease were related to the acceptable indices of the Pan American Health Organization (PAHO)^16^ and the World Health Organization (WHO)^3^. They were then presented on maps, with progressively dark-shaded layers representing increases in incidences. TerraView software (version 4.2.2) was used for the construction of the contiguous neighborhood matrix and the calculation of Bayesian rates, and the QGis software (version 2.18) was used for the construction of thematic maps.

To identify risk areas, the SaTScan scanning technique was used following the application of the discrete Poisson model. This technique allows the identification of areas with a population that is susceptible/non-susceptible to an event through spatial randomness tests^17^. Circular window scanning was selected, using the years as reference time (2008 to 2016). The maximum size of the spatiotemporal cluster was 50% of the population at risk in each area with an average distance between them. The municipalities were represented by 463,579 Cartesian units. Based on the incidences of PS and CS, the SaTScan software (version 9.3) calculated the relative risk (RR) in each municipality by determining the presence of high and low-risk clusters. p-value of 0.05 was fixed as the level of statistical significance, and 999 Monte Carlo replications were considered.

The differences between municipalities belonging to low (0) and high-risk (1) clusters for PS and CS were verified, and these were compared according to sociodemographic and health variables. Municipalities were classified as below median (0) and above median (1) based on the proportion of individuals with inadequate access to household water and sewage services (ratio between people living in households whose water supply did not originate from a general network, with sewage services not provided by a sewerage network or septic tank, and total population residing in permanent private households, multiplied by 100), proportion vulnerable to poverty (proportion of individuals with per capita household income ≤ R$ 255.00 per month, in reals in August 2010, equivalent to 1/2 of the minimum wage on that date), proportion of pregnant women with under 8 years of schooling (ratio between the number of LB mothers with under 8 years of schooling and the total number of LB mothers, multiplied by 100), proportion of pregnant teenagers (aged 19 years or less) (ratio between the number of LB mothers aged 19 years or less and the total number of LB mothers, multiplied by 100), proportion of pregnant women with received seven or more prenatal care sessions (ratio between the number of LB mothers who had seven or more prenatal care and the total number of LB mothers, multiplied by 100), and proportion of LB with a race/non-white color (ratio between number of LB with race/non-white color and the total number of LB, multiplied by 100), as they contain non-parametric data. Pearson’s chi-square (χ^2^) test with a p-value of <0.05 was used to verify the differences between clusters.

Data on the sociodemographic indicators of different municipalities were accessed from the Human Development Atlas of the United Nations Development Program (2010)^18^, and data on LB and the respective mothers were retrieved from the Live Birth Information System (*Sistema de Informação sobre Nascidos Vivos* - SINASC) (2008-2016), the digital network of the Brazilian Institute of Geography and Statistics (IBGE). Data on PS and CS were obtained from the Secretaria de Estado de Saúde de Mato Grosso.

This study met the requirements of Resolution 466/2012 of the National Health Council and was approved by the Ethics Committee of the Escola de Saúde Pública do Estado de Mato Grosso (Opinion Nº 2,178,593 of 07/19/2017 and N° 2,442,202 of 12/15/2017).

## RESULTS

After performing deterministic linkage between data from the SINAN, SIM, and SIH/SUS databases, 3,234 cases of PS and 1,190 cases of CS were identified between 2008 and 2016 in Mato Grosso ( Supplementary Figure 1 **and** Supplementary Figure 2 , respectively).

**FIGURE 1:**
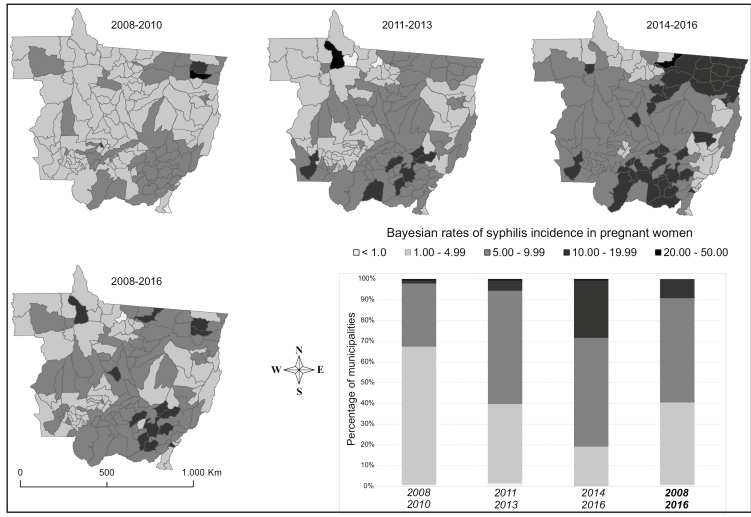
Maps for the distribution of Bayesian rates of syphilis incidence in pregnant women and proportion of municipalities according to incidence strata in each triennial period (2008-2010; 2011-2013; 2014-2016) and during 2008-2016. Mato Grosso, 2008 to 2016. **Incidence:** per 1,000 live births.

During the study period, there was a progressive increase in the incidence of PS in most municipalities of Mato Grosso. In the 2008-2010 triennium, the incidences ranged from 0.9 and 20.5/1,000 LB, with 140 (99.3%) municipalities exhibiting incidences equal to or greater than 1.0/1,000 LB. In the 2011-2013 triennium, the variation ranged from 0.6 to 46.3/1,000 LB, with 139 (98.6%) municipalities exhibiting incidences equal to or greater than 1.0/1,000 LB. In the last triennum (2014-2016), the variation ranged from 2.1 to 23.2/1,000 LB, with 141 (100%) municipalities exhibiting incidences equal to or greater than 1.0/1,000 LB. The number of municipalities with an incidence of PS in the fourth stratum (10-19.9/1,000 LB) increased from two between 2008 and 2010 to 39 between 2014 and 2016 (Figure 1).

**FIGURE 2:**
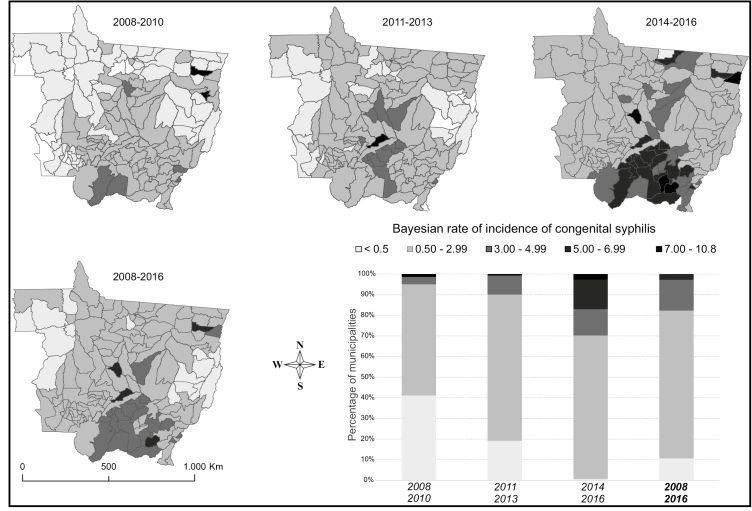
Maps for the distribution of Bayesian rates of congenital syphilis incidence and proportion of municipalities according to incidence strata in each triennial period (2008-2010; 2011-2013; 2014-2016) and during 2008-2016. Mato Grosso, 2008 to 2016. **Incidence:** per 1,000 live births.

A progressive increase in the distribution of CS incidences was also observed. In 2008-2010, 2011-2013, and 2014-2016, the incidences ranged from 0 to 7.1/1,000 LB, 0 to 7.5/1,000 LB, and 0.3 to 10.8/1,000 LB, respectively. The incidence rates in 83 (58.9%), 114 (80.9%), and 140 (99.3%) municipalities were equal to or higher than 0.5/1,000 LB in 2008-2010, 2011-2013, and 2014-2016, respectively. The number of municipalities with CS incidence in the fourth stratum (5.1-7.0/1,000 LB) increased from one municipality in the first triennium to 19 municipalities in the last triennium (Figure 2).

**FIGURE 3:**
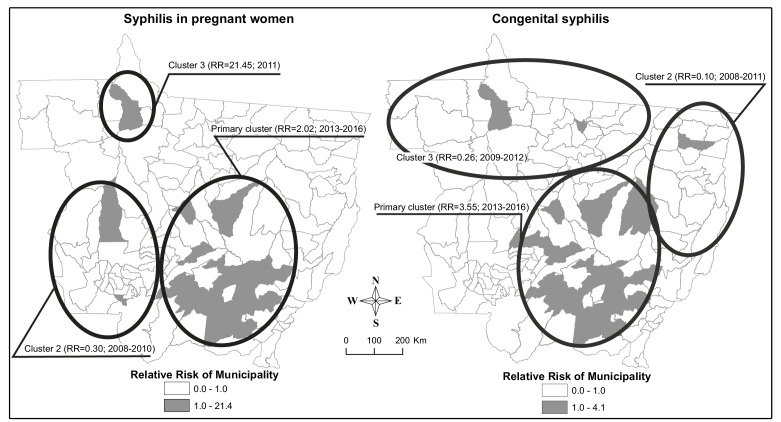
Relative risk maps and spatiotemporal clusters (high and low rates) of syphilis in pregnant women and congenital syphilis. Mato Grosso, 2008-2016. **RR:** relative risk.

In the SATSCAN analysis, three statistically significant clusters were identified for both PS and CS. The primary PS cluster (RR=2.02; p <0.0001) located in the southern, southeastern, and central regions of Mato Grosso, consisted of 34 municipalities, and 1,231 cases from 2013 to 2016. The second cluster (RR=0.30; p <0.0001) consisted of cases reported in the 24 municipalities located in the western and southwestern regions of the state from 2008 to 2010, including 45 cases of PS. The third cluster (RR=21.45; p <0.0001) was for cases reported in 2011 in Nova Bandeirantes city, in the northwestern region of the state, where 22 PS cases were reported (Figure 3). The primary cluster for CS (RR=3.55; p <0.0001) included 618 cases and was superimposed with respect to time and space over the primary PS cluster. Both primary clusters reported the highest occurrence of cases. The second cluster for PS (RR=0.10; p <0.0001), for cases reported in the northern and northwestern regions between 2008 and 2011, comprised 26 municipalities and reported seven cases. The third cluster (RR=0.26; p = 0.027) consisted of cases reported between 2009 and 2012 in the eastern and northeastern regions of Mato Grosso and comprised eight cases of CS distributed across 22 municipalities (Figure 3).

With respect to PS, the high-risk cluster consisted of municipalities with the proportion of pregnant women with under 8 years of schooling below median. For CS, the high-risk cluster had a higher proportion of municipalities with inadequate water supply and sewage and LB mothers with a race/non-white color above median. Furthermore, the high-risk cluster for CS was characterized by municipalities with higher proportion of pregnant women with under 8 years of schooling, and proportion of pregnant teenagers below median (Table 1).

**TABLE 1:** Differences between low and high-risk clusters for congenital syphilis and syphilis in pregnant women according to sociodemographic data and health indicators. Mato Grosso, 2008 to 2016

Indicators	Municipalities belonging to clusters							
	Syphilis in Pregnant Woman				Congenital Syphilis			
	Total	low risk	high risk	p-value*	Total	low risk	high risk	p-value*
	58	24 (%)	34 (%)		82	48 (%)	34 (%)	
**PHISF**				0.563				0.001*
< median	24	11 (45.8)	13 (38.2)		49	36 (75.0)	13 (38.2)	
≥ median	34	13 (54.2)	21 (61.8)		33	12 (25.0)	21 (61.8)	
**VP**				0.825				0.146
< median	28	12 (50.0)	16 (47.1)		31	15 (31.3)	16 (47.1)	
≥ median	30	12 (50.0)	18 (52.9)		51	33 (68.7)	18 (52.9)	
**PWS (< 8 years)**				0.032*				<0.001*
< median	36	11 (45.8)	25 (73.5)		39	14 (29.2)	25 (73.5)	
≥ median	22	13 (54.2)	9 (26.5)		43	34 (70.8)	9 (26.5)	
**PWT (≤ 19 years)**				0.231^$^				<0.001*
< median	43	20 (83.3)	23 (67.6)		36	13 (27.1)	23 (67.7)	
≥ median	15	4 (16.7)	11 (32.4)		46	35 (72.9)	11 (32.3)	
**LBPC (≥ 7)**				0.397				0.628
< median	30	14 (58.3)	16 (47.1)		36	20 (41.7)	16 (47.1)	
≥ median	28	10 (41.7)	18 (52.9)		46	28 (58.3)	18 (52.9)	
**LBRC non-white**				0.263				0.040*
< median	24	12 (50.0)	12 (35.3)		40	28 (58.3)	12 (35.3)	
≥ median	34	12 (50.0)	22 (64.7)		42	20 (41.7)	22 (64.7)	

Pearson's ᵡ^2^ test; ^$^Fisher’s exact test. *p-value <0.05 was considered statistically significant. **PHISF:** % people in homes with inadequate sanitation facilities; **VP:** % vulnerable to poverty; **PWS (< 8 years):** % pregnant woman < 8 years of schooling; **PWT (≤ 19 years):** % pregnant woman teenager (≤ 19 years); **LBPC (≥ 7):** % LB with ≥ 7 prenatal care; **LBR non-white:** % LB race/non-white color. **Median:** PHIBW = 62.88%; VP = 33.87%; PWS (< 8 years) = 29.84%; PWT (≤ 19 years) = 23.96%; LBPC (≥ 7) = 68.39%; LBRC non-white = 69.75%. **Note:** The high-risk cluster for PS in Nova Bandeirantes was excluded from this analysis because it was a single-year event (2011).

## DISCUSSION

The findings indicate an increase in disease incidences in most municipalities in Mato Grosso during the study period, as well as the presence of higher risk areas for PS and CS.

The spatiotemporal evolution of PS and CS incidences in Mato Grosso corroborate the findings from other studies conducted in the country^19^ ^,^ ^20^, as well as the increase in syphilis incidence rates observed in recent years in Brazil^6^. The increase was attributed by the Ministry of Health to improvements in health information systems, greater number of opportunities for rapid testing in Basic Health Units (UBS), reduced condom use, decreased benzathine penicillin stocks, and the reservations among health professionals regarding the administration of this antibiotic in UBS^4^.

The local empirical Bayesian model, with spatiotemporal dependence between neighboring areas, allowed the identification of spatial variation and increasing trends of the temporal evolution of CS and PS incidences in the municipalities of Mato Grosso. A progressive increase was observed in the proportion of municipalities associated, especially in the strata with higher incidence rates for both forms of the disease. In the last three years of the study, the incidences of PS and CS were higher than the rates recommended by PAHO^7^ and WHO^3^ in all municipalities except Guarantã do Norte, in the northeastern region of the state (only with respect to the incidence of CS).

Although the incidence of PS increased across the state during the study period, municipalities in the central-southern, southeastern, and northeastern regions of Mato Grosso had the highest rates. With respect to the spatial distribution of CS, an incidence expansion was also observed, and in the last three years, the highest rates of incidence led to the establishment of an “extensive syphilis corridor” from the south-central and southeastern regions to the northern region of the state. Mato Grosso is the largest beef cattle producer in the country and is the primary national grain producer, with the northern and central-southern regions as the predominant producers. State highways have formed the primary thoroughfare for these agricultural products^21^, and the majority of economic development and population growth in Mato Grosso has occurred along the margins of these highways^22^. The development of a region owing to the establishment of highways can contribute to the introduction, dissemination, or reduction of diseases through changes in the social and natural environment^23^ ^,^ ^24^.

The south-central and southeastern regions of Mato Grosso have an important industrial food center, along with mineral extraction sites in several regions, which may contribute to migration and fluctuating population in these locations. In addition, the northern and northeastern regions of Mato Grosso are bordered by the states of Tocantins and Pará, and their capital cities Palmas and Belém, respectively, had higher incidences of PS and CS than the national average of Brazil in 2017^6^. In the same year, Tocantins also reported the highest infant mortality rate caused by CS (33.5/100,000 LB) in the country^6^.

Studies conducted in the areas of influence in Brazil’s Cuiabá-Santarém (BR-163) highway, Transamazon (BR-230) highway^24^, and Brazil’s international borders^25^ have indicated the major factors such as migration, drug use, the presence of a floating male population, and disruption of social structures that promote sexual trade and exacerbate the transmission of human immunodeficiency virus (HIV). In a study by Barcellos et al.^24^, high incidence rates of acquired immune deficiency*syndrome* (AIDS) were related to the increase in GDP and to high proportions of the urban population living in municipalities belonging to the BR-163 area of influence in the route between Cuiabá-Santarém and the Transamazon highway (BR-230).

The low-risk areas for incidences of PS and CS identified in the early years of the study declined in subsequent years, while high-risk areas emerged during the latter years of study. These findings show the rapid and intensive spatial progression of these two diseases in the municipalities of Mato Grosso between 2008 and 2016. The presence of overlapping high-risk clusters of PS and CS is distinct and persisted in the last four years of the study in the southern, southeastern, and central regions of Mato Grosso. Temporal and geographical persistence refers to the continuity and expansion of transmission, which may be suggestive of the social vulnerability of these geographical areas. The overlapping of high-risk clusters reflects possible difficulties in accessing health services, failures in the treatment of maternal infection, and inadequate prevention of mother-to-child transmission of syphilis in the municipalities involved, which suggests poor assistance to pregnant women and maternity. International studies have revealed an overlap of high-risk syphilis clusters with those of other sexually transmitted infections (STIs)^26^ ^-^ ^28^. As this study did not aim to verify this occurrence, further studies on the spatialization of syphilis should include other STIs to provide additional information for epidemiological surveillance.

In this study, the high-risk clusters of CS and PS were characterized by municipalities with the poorest sanitary conditions and higher proportion of LB mothers with a race/non-white color, which revealed the inequalities in the distribution pattern of syphilis in Mato Grosso. However, the analysis of secondary ecological data does not allow inference at the individual level. Moreover, syphilis is an infection that has been associated with the human race since its appearance before recorded history and is related to biological activities common to all individuals, such as sexual practice. Possibly owing to this reason, it was difficult to identify a pattern that could indicate the spread of PS and CS in Mato Grosso along with the social, economic, and mobility factors of the population. Apparently, incidences of PS and CS increased later in areas distant from major road junctions and with less economic development. Nevertheless, the findings of this study show that syphilis can spread beyond social boundaries or conditions, and even educated populations are at risk of infection.

Affective and sexual behavior is an essential and intimate aspect of human beings, and is characterized by habits, preferences, and taboos that can be difficult to address or control. Furthermore, it should be emphasized that syphilis is often asymptomatic, which makes detection and control challenging. Therefore, the increasing dissemination of PS and CS in multiple countries can only be controlled by implementing health education measures.

The use of secondary data was one of the limitations of this study. However, the linkage between different PS and CS data sources contributed to the estimation of real case numbers.

The findings of this study clearly indicate the increasing evolution of PS and CS in Mato Grosso and its relevance to public health. The analysis revealed the persistence of high-risk clusters, which indicated a higher incidence of *Treponema* circulation in certain municipalities and the need for its control. Health authorities should be advised to direct their efforts to (i) monitor maternal and congenital syphilis, especially in areas identified as critical areas; (ii) promote training and awareness of health teams regarding the reporting of these infections in SINAN and other health information systems; (iii) ensure quality prenatal care with early pregnancy uptake, access to early diagnosis (rapid testing for syphilis and other STIs), and appropriate treatment.
