# Supplementary figures and images for: Syphilis in pregnant women and congenital syphilis: spatial pattern and relationship with social determinants of health in Mato Grosso
Source: Rev Soc Bras Med Trop. 2020 Oct 21;53:e20200316. doi: 10.1590/0037-8682-0316-2020 (PMC7580285; doi:10.1590/0037-8682-0316-2020)

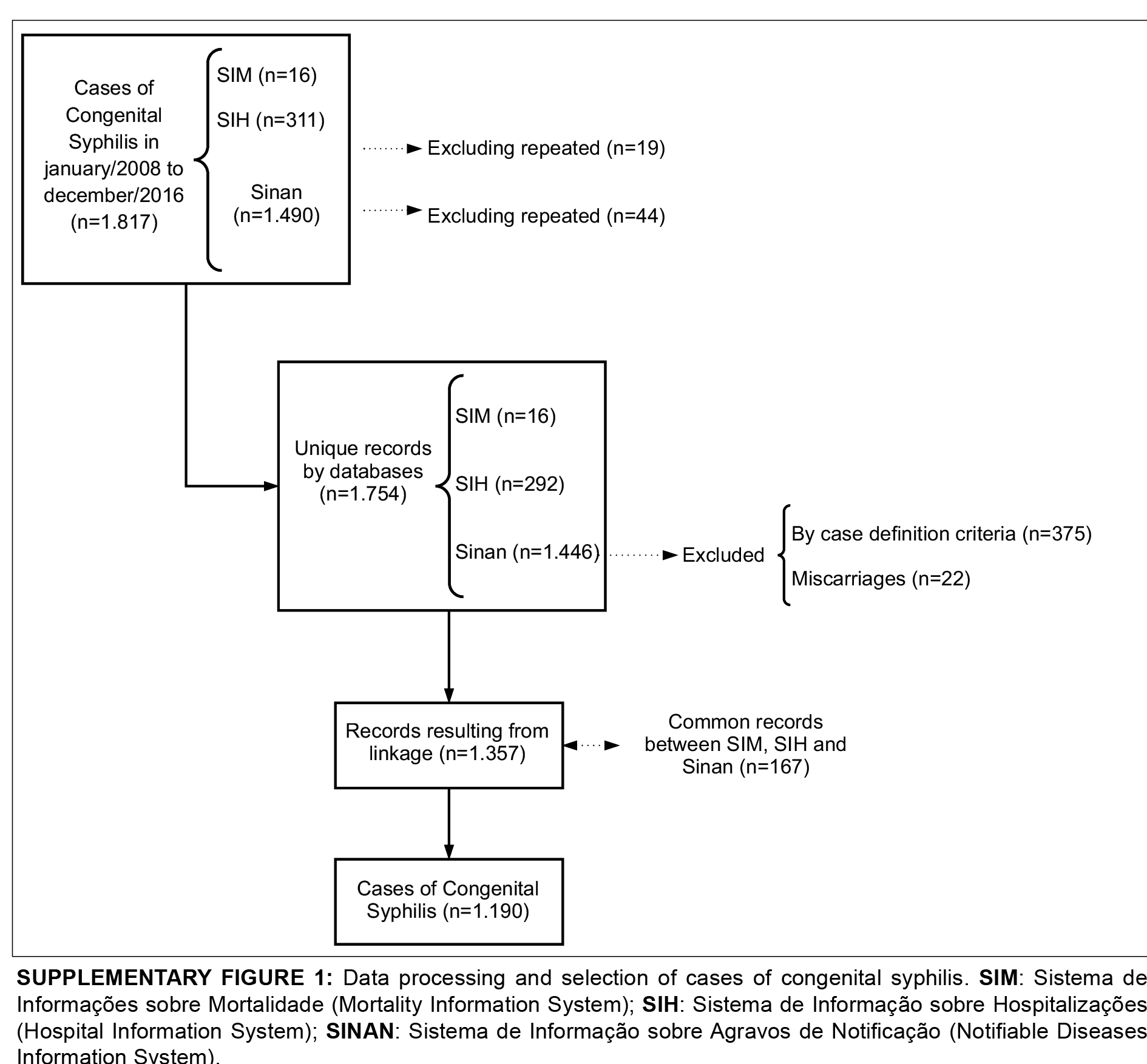

Supplement: Supplementary file 1 [file 1678-9849-rsbmt-53-e20200316-suppl1.jpg]

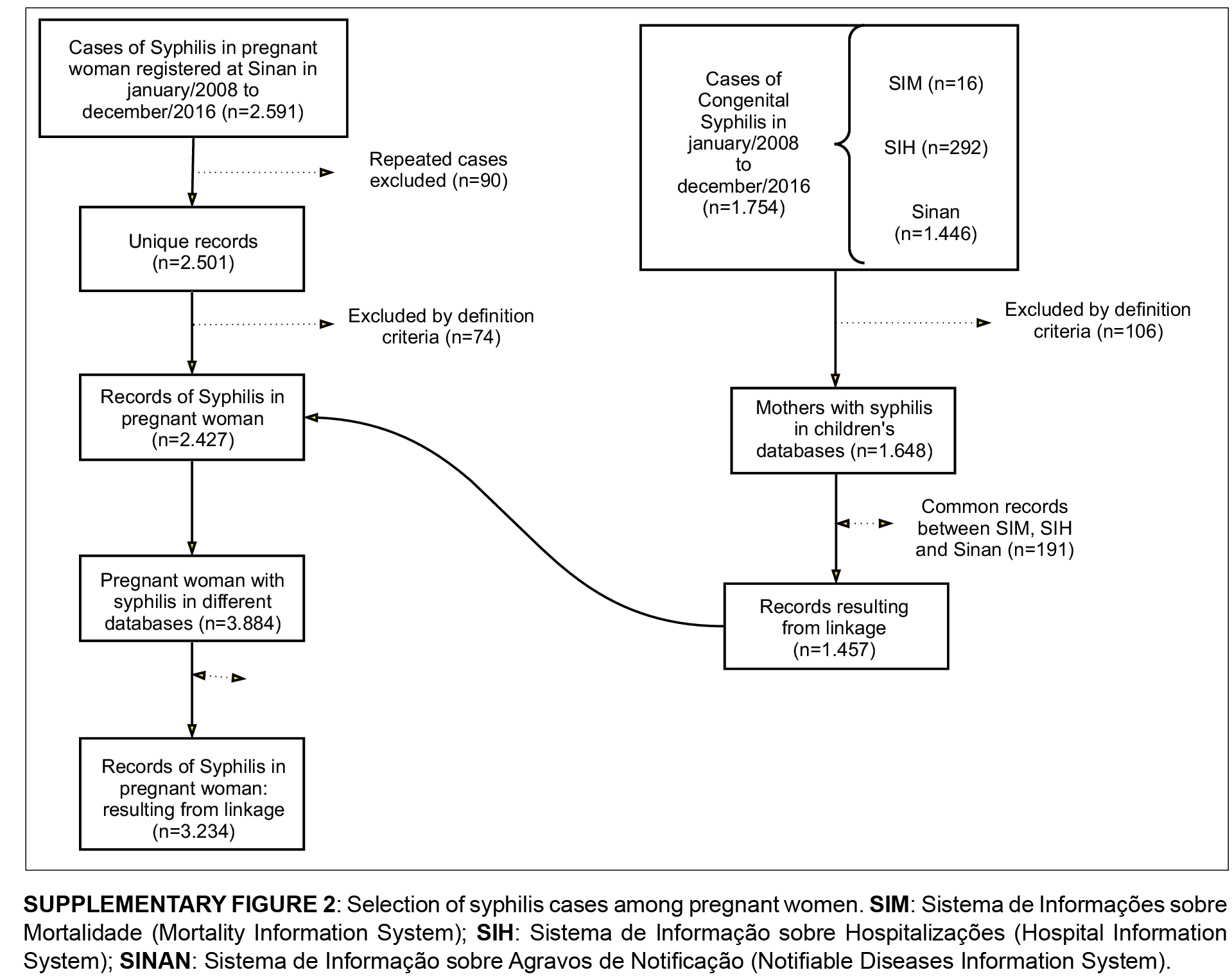

Supplement: Supplementary file 2 [file 1678-9849-rsbmt-53-e20200316-suppl2.jpg]
